# GLI2 inhibition abrogates human leukemia stem cell dormancy

**DOI:** 10.1186/s12967-015-0453-9

**Published:** 2015-03-21

**Authors:** Anil Sadarangani, Gabriel Pineda, Kathleen M Lennon, Hye-Jung Chun, Alice Shih, Annelie E Schairer, Angela C Court, Daniel J Goff, Sacha L Prashad, Ifat Geron, Russell Wall, John D McPherson, Richard A Moore, Minya Pu, Lei Bao, Amy Jackson-Fisher, Michael Munchhof, Todd VanArsdale, Tannishtha Reya, Sheldon R Morris, Mark D Minden, Karen Messer, Hanna KA Mikkola, Marco A Marra, Thomas J Hudson, Catriona HM Jamieson

**Affiliations:** Department of Medicine, Stem Cell Program and Moores Cancer Center, University of California San Diego, 3855 Health Sciences Drive, La Jolla, 92037 CA USA; Canada’s Michael Smith Genome Sciences Center, British Columbia Cancer Agency, Vancouver, BC Canada; Eli and Edythe Broad Center for Regenerative Medicine and Stem Cell Research, University of California, Los Angeles, CA USA; Ontario Institute for Cancer Research, Toronto, ON Canada; Department of Medicine, University of Toronto, Toronto, ON Canada; Princess Margaret Cancer Center, University Health Network, Toronto, ON Canada; Pfizer, La Jolla, CA USA; Division of Regenerative Medicine, University of California San Diego, 3855 Health Sciences Drive, La Jolla, CA 92093-0820 USA

**Keywords:** Leukemia stem cells, Cell cycle, PF-04449913, Sonic hedgehog, Smoothened SMO, GLI2

## Abstract

**Background:**

Dormant leukemia stem cells (LSC) promote therapeutic resistance and leukemic progression as a result of unbridled activation of stem cell gene expression programs. Thus, we hypothesized that 1) deregulation of the hedgehog (Hh) stem cell self-renewal and cell cycle regulatory pathway would promote dormant human LSC generation and 2) that PF-04449913, a clinical antagonist of the GLI2 transcriptional activator, smoothened (SMO), would enhance dormant human LSC eradication.

**Methods:**

To test these postulates, whole transcriptome RNA sequencing (RNA-seq), microarray, qRT-PCR, stromal co-culture, confocal fluorescence microscopic, nanoproteomic, serial transplantation and cell cycle analyses were performed on FACS purified normal, chronic phase (CP) chronic myeloid leukemia (CML), blast crisis (BC) phase CML progenitors with or without PF-04449913 treatment.

**Results:**

Notably, RNA-seq analyses revealed that Hh pathway and cell cycle regulatory gene overexpression correlated with leukemic progression. While lentivirally enforced GLI2 expression enhanced leukemic progenitor dormancy in stromal co-cultures, this was not observed with a mutant GLI2 lacking a transactivation domain, suggesting that GLI2 expression prevented cell cycle transit. Selective SMO inhibition with PF-04449913 in humanized stromal co-cultures and LSC xenografts reduced downstream GLI2 protein and cell cycle regulatory gene expression. Moreover, SMO inhibition enhanced cell cycle transit and sensitized BC LSC to tyrosine kinase inhibition in vivo at doses that spare normal HSC.

**Conclusion:**

In summary, while *GLI2*, forms part of a core HH pathway transcriptional regulatory network that promotes human myeloid leukemic progression and dormant LSC generation, selective inhibition with PF-04449913 reduces the dormant LSC burden thereby providing a strong rationale for clinical trials predicated on SMO inhibition in combination with TKIs or chemotherapeutic agents with the ultimate aim of obviating leukemic therapeutic resistance, persistence and progression.

**Electronic supplementary material:**

The online version of this article (doi:10.1186/s12967-015-0453-9) contains supplementary material, which is available to authorized users.

## Background

Chronic myeloid leukemia (CML) is a myeloproliferative disease arising from the acquisition of the Philadelphia chromosome (Ph) by hematopoietic stem cells (HSC). This is characterized by the reciprocal translocation between the long arms of chromosomes 9 and 22, t (9;22) (q34;q11) which in turn gives rise to a fusion of c-ABL and BCR to generate the BCR-ABL fusion gene that encodes the constitutively active tyrosine kinase BCR-ABL1 [1]. This results in the modulation of several key-signaling cascades leading to increased cell proliferation, induction of cellular transformation and blockage of apoptotic pathways. In CML, chronic phase occurs after BCR-ABL1 oncogene expression in hematopoietic stem cells (HSCs), that can then generate an expansion of a pool of myeloid GMP (CD34 + CD38 + Lin^−^) progenitors that aberrantly acquire the capacity to self-renew, leading to progenitor LSC generation and BC transformation [2].

Deregulation of critical stem cell processes, such as self-renewal and dormancy, in hematopoietic progenitors contributes to the generation of leukemia stem cells (LSC) that are impervious to therapies that target dividing cells [2-14]. In advanced human myeloid malignancies that progress from antecedent hematologic malignancies, such as blast crisis (BC) CML, LSC have a proclivity for becoming dormant in protective niches [2,5,7,11,15-18].

Targeted therapies for the treatment of CML using tyrosine kinase inhibitors (TKI) that block BCR–ABL activity have advanced in recent years to include not only imatinib but also second-generation TKIs such as dasatinib and nilotinib. While a striking reduction in CML-related mortality has occurred in patients treated early in chronic phase with BCR-ABL1 targeted tyrosine kinase inhibitor (TKI) therapy, the majority of patients relapse with detectable BCR-ABL1 transcripts following TKI discontinuation, which has been attributed to persistence of dormant BCR-ABL expressing hematopoietic stem and progenitor cells [19,20] Compelling studies, such as the STIM trial, in which a majority of CML patients relapsed within 12 months of TKI discontinuation, suggest that new therapeutic strategies will be required to achieve molecular cures [21].

Previous studies have established a role for aberrant activation of Hh signaling in a number of solid tumors as well as hematologic malignancies [16,17]. Hh proteins (ligands) bind to the receptor patched (PTCH), causing the release of inhibition by smoothened (SMO) and the subsequent activation of transcription factors GLI-1 and GLI-2 that then translocate to the nucleus and activate their target genes [22]. As the main effectors of Hh pathway signaling following SMO activation, the GLI transcriptional factors, GLI1 and GLI2 [23,24] promote oncogenesis through up-regulation of genes encoding apoptosis inhibitors (e.g., Bcl-2, cFLIP) [25,26] and angiogenesis inducers (e.g., vascular endothelial growth factor) [27-33]. Thus, GLI down-regulation through SMO antagonism could reduce LSC maintenance driven by both Hh ligand dependent and ligand independent epigenetic activation mechanisms [34,35].

In CML Hh signaling is vital for maintenance of LSC through SMO-mediated GLI1 activation but in humans GLI2 increases with blastic transformation [36]. Moreover, activation of the Hh stem cell pathway agonist, smoothened (SMO) and downstream glioma associated (GLI) zinc finger transcriptional regulators, promoted LSC maintenance in a mouse model of CML [17]. Previously, we uncovered a GSK3β missplicing event that prevented degradation of both β-catenin and GLI transcription factors in serially transplantable LSC [7]. Others studies have analyzed newly diagnosed cases of CML and found differential activation of Sonic Hh (SHH) signaling was seen in 50% of chronic phase, 70% of accelerated phase and over 80% of blast crisis cases. It was shown that auto-activation of SHH signaling provided survival and proliferative cues in CML progenitor cells through downstream β- catenin signaling and that by blocking the pathway with inhibitor or neutralizing antibodies, apoptosis could be induced [37].

While Hh is involved in embryonic pattern formation, including the hemogenic endothelial to hematopoietic transition [38] it also plays a pivotal role in the maintenance of adult neural and skin stem cell populations. However Hh signaling may be dispensable for maintenance of normal adult hematopoiesis [39-41] suggesting that a therapeutic window may exist for targeting human LSC compared with normal HSC and progenitor populations in the bone marrow and other hematopoietic niches.

Despite extensive transcriptional profiling of BC CML samples [11,13,14], the cell type and context specific role of Hh pathway activation in human LSC therapeutic resistance and cell cycle deregulation had not been elucidated. Moreover, diagnostic strategies that predict progression and therapies capable of abrogating human LSC dormancy while sparing normal HSC function have remained elusive. In this study, we performed RNA-seq, humanized stromal co-cultures, human LSC xenograft assays, lentiviral overexpression and multi-parameter cell cycle FACS analysis to investigate whether 1) Hh pathway and dormancy gene activation are biomarkers of myeloid leukemic progression and LSC generation; 2) to determine if GLI2 drives leukemic progenitor cell cycle deregulation in human LSC supportive niche assays and 3) if selective inhibition of SMO by PF-04449913 reduces niche-dependent LSC maintenance and therapeutic resistance in LSC in vivo models.

## Methods

### Bone marrow and peripheral blood banked samples

Normal cord blood and adult peripheral blood samples were purchased from All Cells. Myeloid malignancy peripheral blood and bone marrow samples were obtained from consenting patients enrolled in Institutional Review Board approved protocols. Human hematopoietic stem and progenitors cells were FACS Aria purified as described previously [42].

### Whole transcriptome RNA sequencing

Progenitor cells (n = 50,000) derived from viably cryopreserved normal cord blood (n = 3), normal peripheral blood (n = 3), CP CML (n = 8) and BC CML (n = 9) samples, were FACS Aria sorted directly into RLT buffer (Qiagen) and LSC microarray studies were performed as described previously [42]. For each sample, approximately 5 ng of total RNA was processed using smarttm cDNA synthesis protocol with smartscribe reverse transcriptase (clontech, #639536), which used a modified oligo (dt) primer to prime the first strand synthesis reaction and a template switching mechanism to generate full length single stranded cDNAs containing the complete 5’ end of the mRNA as well as universal priming sequences for end-to-end amplification by 20 cycles of PCR. The resultant cDNAs were fractionated and size-selected for fragments with 250-350 bp in length. The amplified cDNA was subjected to Illumina paired end library construction using NEBNext paired-end DNA sample Prep Kit (NEB, E6000B-25). Libraries sequenced on Illumina HiSeq2000 instruments with the average of approximately 163.8 million Chastity-passed read pair with 50 bp in read length. Spearman’s rank correlation coefficient was used as the distance metric. To test whether the grouping in the PC plots was statistically significant, permutation tests were performed by random sampling of gene sets with a sample size equal to our signature gene set from KEGG-annotated genes in MSigDB. Genes were excluded if expression levels were zero across all subjects; a total of 4617 genes remained. We drew 5000 random sets in total. A principal components analysis was performed on each random data set and an MANOVA model was built using the first 3 principal components as response variables and the group label as the predictor. A permutation p-value was defined as the proportion of the F-statistic from the original data set that was greater than the F-statistics from the random sets.

Illumina platform RNA-seq read alignment, coverage analysis and clustering: Using BWA software (version 0.5.7) [43], the Chastity-passed reads were aligned to the human reference genome (hg19/GRch37) plus exon junction sequences constructed from all known transcript models in RefSeq, EnsEMBL and known genes from the UCSC database as described previously [44]. We used default parameter settings of BWA except for the option –s to disable Smith-Waterman alignment. After the alignment, the reads that aligned to exon junctions were repositioned in the genome as large-gapped alignments using repositioning software developed in-house. We removed adapter dimer sequences and soft-clipped reads that contained adapter sequences. The unambiguously aligned, filtered reads were then analyzed by in-house developed gene coverage analysis software to calculate the coverage over the total collapsed exonic regions in each genes as annotated in EnsEMBL (version 59), and the RPKM values were calculated to represent the expression level of exons and genes. We used the raw read counts for differential gene expression analysis using DESeq (v1.4.1) in R. The multiple hypothesis testing correction of p-values from Mann–Whitney *U* test was done using Benjamini-Hochberg method in p.adjust function in R. We performed unsupervised hierarchical clustering using complete linkage on log2-transformed RPKM values that were centered on the median. Spearman’s rank correlation coefficient was used as the distance metric for clustering samples and genes. The clustering results were visualized using Treeview (Java Cluster software). We used RPKM values and performed the non-negative matrix factorization clustering using NMF (v0.5.06), Biobase (v2.14.0) and cluster (v1.14.2) packages in R. We ran the NMF clustering using the default ‘brunet’ method for the NMF algorithm and set the seeding method to default ‘random’ and performed the clustering over 200 iterations. For the factorization rank survey, we performed clustering using 50 iterations for our dataset, and 25 iterations for a randomized dataset. The visualization of the consensus matrix heatmap and the cophenetic correlation coefficient plot was done using the plot function in R.

### Transcriptome analysis of LSC isolated from PF-04449913 and vehicle treated mice

### Sample preparation, library construction and sequencing

Neonatal RAG2−/−c−/− mice transplanted intrapheptically with 50,000 BC CML LSC were treated 8 weeks later with vehicle or PF-04449913 (100 mg/kg) for 2 weeks by oral gavage. Four mice were treated with vehicle and four mice were treated with SMO inhibitor (Additional file 1: Table S3). Mice were sacrificed and human leukemia stem cells (~50,000 cells/sample) were sorted from the liver into RLT buffer from the Qiagen Rneasy kit and RNA was extracted. Total RNA samples were treated once with Ribominus kit (Invitrogen, #A10837-08) to deplete ribosomal RNA. From the resulting RNA whole transcriptome libraries were prepared for SOLiD sequencing. Samples were sequenced in 2 different batches to produce 50 bp fragment (i.e. non-paired-end) reads with an average of approximately 118 million reads. Samples 1–7 were sequenced with SOLiD v3.5 chemistry and samples 8–12 were sequenced SOLiD v4 chemistry.

The limma method was used to test for main effects of PF-04449913 and Dasatinib, and their synergistic interaction among 41 genes. Null hypotheses were rejected at p = 0.05 significance level without adjusting for multiple comparisons.

### GSEA analysis

We filtered RNA Seq data using the raw read counts per gene to included genes with at least 10 mapped reads in one or more samples. A total of 13,850 protein-coding genes were included in the analysis. The counts were normalized using upper-quartile normalization. Significance Analysis of Microarrays (SAM) was used to rank the genes according to their differences in expression levels between the four SMO inhibitor treated mice and the four vehicle mice. Gene set enrichment analysis (GSEA) was used to assess the effect of SMO treatment on cell cycle pathways. Of the eight a priori cell cycle pathways considered in the analysis, “Regulation of Cell Cycle” was significantly down regulated comparing treated mice (n = 4) to control mice (n = 4) (family-wise p value =0.02). Six additional pathways out of the 8 total were observed to be down regulated, although not significantly. Table columns are pathway name, number of genes included in the pathway, nominal p-value, FDR adjusted q-value and adjusted p-value controlling for the family-wise error rate. Initially, all 13,850 genes were ranked according to SAM score, and from this ranking the GSEA score for the pathway was computed. The significance of the GSEA score was assessed by randomly permuting gene labels 2000 times and a family wise p-value was computed, correcting for the 8 pathways tested.

*For the GSEA enrichment plot for the down*-*regulated* “*Regulation of Cell Cycle*” *Pathway*, *a*ll 13,850 genes were ranked in descending order based on the Significance Analysis of Microarrays (SAM) score comparing four treated mice vs. four control mice (horizontal heatmap). The GSEA enrichment score for the pathway (genes indicated as vertical bars) is indicated as the maximal excursion of the green line. This pathway has 41 genes; however one gene was filtered out due to low expression. Because the pathway is down regulated, the core enrichment subset consists of the right-most 18 among the 41 expressed genes in the pathway.

### Quantitative PCR analysis

The following primers were used in reactions run with SYBR: BCR-ABL Forward: ctccagactgtccacagcat, BCR-ABL Reverse: ccctgaggctcaaagtcaga, HPRT Forward: cgtcttgctcgagatgtgatg, HPRT Reverse: tttatagccccccttgagcac. Relative levels of mRNA were determined according to standard curves. All values were then normalized to HPRT or RPL27 values from the same sample. TaqMan primer/probe sets (ABI) for FoxM1 (Mm00514924_m1), Gli1 (Mm00494645_m1), Gli2 (Mm01293117_m1), Mycn (Mm00476449_m1), Ptch1 (Mm00436026_m1), Ptch2 (Mm00436047_m1), Sfrp1 (Mm00489161_m1), Smo (Mm01162710_m1) on the ABI 7900HT instrument. Target gene expression levels were normalized to GAPDH and to vehicle treated mice to yield the relative quantitation (RQ) value. Expression qRT-PCR arrays (SA biosciences) were also utilized to quantify SHH pathway gene expression.

### Stromal co-culture assays

Normal or BC CML CD34^+^ cells were plated on confluent mitomycin-C treated SL/M2 cells with vehicle, PF-04449913 (1uM), dasatinib (50nM), or combination treatment. Mouse bone marrow stromal cell lines, M2-10B4 (M2) and SL/SL (SL) (Stem Cell Technologies Inc, Vancouver, British Colombia) were treated with mitomycin-C (1 mg/ml) and plated in a 1:1 mixture at a total concentration of 100,000 cells/ml one day prior to co-culture with 10,000-20,000 CD34+ BC CML or normal progenitors. After 1 week of culture, progenitors were FACS sorted into hematopoietic progenitor assays and colonies were scored at 14 days as previously described [14]. To assess survival of normal human hematopoietic stem and progenitor cells, irradiated (20 Gray) OP9 (M2 clone) stromal cells were co-cultured with 50,000 human CD34^+^ cord blood cells, vehicle or PF-04449913 in AlphaMEM (Gibco) with 20% Hyclone FBS, 1% pen strep glutamine and supplemented with 50 ng/ml SCF, 10 ng/ml thrombopoietin, and 10 ng/ml Flt3 and quantified by weekly FACS analysis.

### Normal and leukemia stem cell xenograft assays

Neonatal immunocompromised RAG2^−/−^c^−/−^ mice were transplanted intrahepatically with equal numbers of normal progenitors or BC LSC as previously described [42]. Upon detection of human CD45^+^ cell peripheral blood engraftment, mice were treated daily by oral gavage with vehicle (50% 1,2 Propandiol, 50% HBSS or methylcellulose), PF-04449913 (100 mg/kg), Dasatinib (50 mg/kg), or the combination for 14 days followed by FACS to quantify human engraftment in hematopoietic niches. To assess effects on normal HSC function, 7 to 10 week old NOD.Cg-PrkdcSCID IL2R1Wjl/SzJ mice were sublethally irradiated, transplanted retro-orbitally with 100,000 CD34^+^ human cord blood cells and treated 8 weeks later with vehicle or PF-04449913 (100 mg/kg) for 14 days followed by FACS engraftment analysis.

### Confocal fluorescence microscopic and nanoproteomic GLI2 analysis

Human BC LSC xenografted mouse spleens were embedded in OCT freezing media (Sakura, Torrance, CA) frozen at −80°C, sectioned and stained with anti-GLI2 (Sigma) and Alexa 647-conjugated anti-human CD45 (1:25, Serotec) antibodies followed by mounting with Prolong® Gold antifade with DAPI (Invitrogen). Confocal fluorescence images were acquired using an Olympus Fluoview FV10i microscope. Nanofluidic phospho-proteomic immunoassay (NPI) experiments were performed in triplicate with the Nanopro 1000 instrument (Cell Biosciences). After separation and photo-activated in-capillary immobilization, GLI2 was detected using a GLI2-specific antibody (Abcam). A β2-microglubulin-specific antibody (β2M; Upstate) was used to normalize the amount of loaded protein. The peaks were quantified by calculating the area under the curve (AUC).

### Cell cycle FACS analysis

Single cell suspensions of bone marrow cells from LSC xenografted mice treated with vehicle or PF-04449913, were immunostained with Alexa647-conjugated anti-human CD45 (BioLegend) and the LIVE⁄DEAD® stain (Invitrogen). Cells were then immunostained with FITC-conjugated anti-Ki-67 (Abcam, 1:100) in 0.15% saponin/2% fetal bovine serum/PBS and incubated with 7- AAD (10 μg/mL in 0.1 M sodium citrate/5 mM EDTA pH8.0/0.15 M NaCl/0.5% BSA/0.02% saponin) followed by FACS Aria analysis.

### Statistical analysis

Statistical analyses for pre-clinical studies were performed with Microsoft Excel and Graphpad Prism software. Continuous variables for each comparison group were assessed for distribution through univariate statistics. If the assumption of normal distribution could be supported, then the Student’s *t* test was performed for comparison of two samples with assessment of equality of variance with an F statistic. If the assumption of normal distribution was not supported, nonparametric testing was performed with the two samples Wilcoxon test using the *t* approximation for samples with N of less than 20. Differential gene expression from RNA-Seq data was detected using R package, DESeq, (v1.4.1) with a significance threshold at 5% false discovery rate (FDR). The Wilcoxon test was used for comparing means of genes expression and identifying genes with significant fold changes. P-values were corrected for multiple hypotheses testing using the Benjamini-Hochberg method. Unsupervised hierarchical clustering analysis was performed using complete linkage on log2-transformed RPKM values that were centered on the median.

## Results

### Hh pathway deregulation signifies human leukemic progression and LSC generation

**Figure 1:**
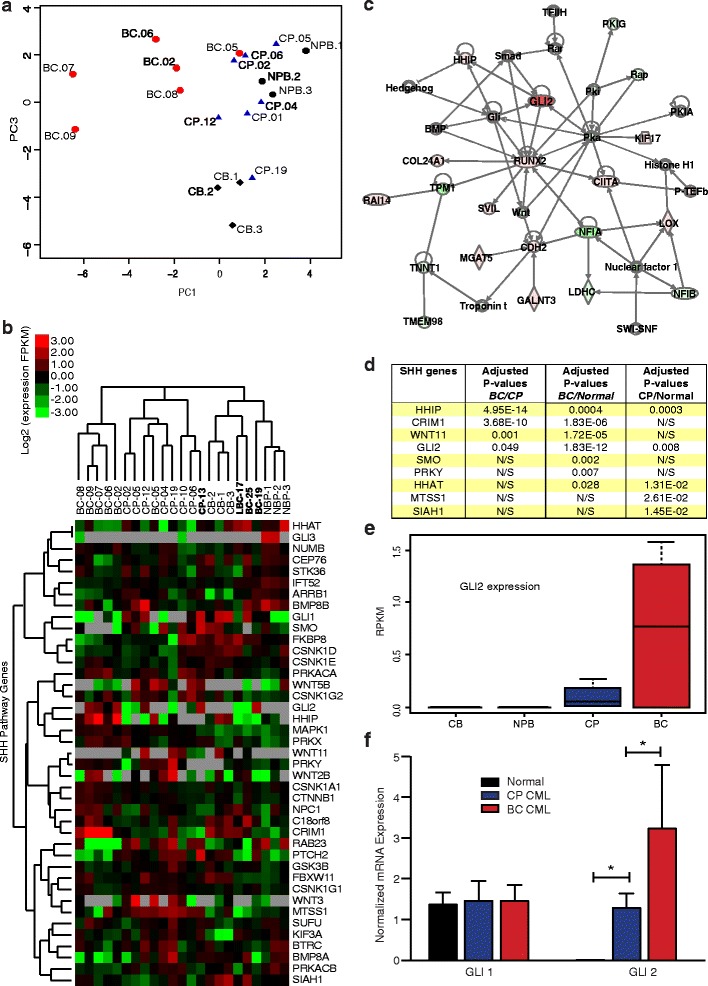
**SHH pathway deregulation in chronic myeloid leukemia progression. a**. Principal components plots derived from RNA-Seq data for 41 genes in the SHH pathway, from 7 chronic phase (CP; blue triangles) and 6 blast crisis (BC; red circles) untreated subjects, as well as 3 cord blood normal samples (CB; black diamonds) and 3 normal peripheral blood (NPB; black circles). **b**. Heatmap from unsupervised agglomerative hierarchical clustering of sonic hedgehog (SHH) pathway genes using RNA-Seq data from FACS-purified progenitors (CD34^+^CD38^+^lin^−^PI^−^) from 8 chronic phase (CP) and 9 blast crisis (BC) patients, 3 normal cord blood (CB) and 3 normal peripheral blood (NPB) sample. Samples labeled in bold correspond to patients that received clinical BCR-ABL inhibitor therapy. Red indicates over- and green, under-expression relative to the median RPKM (Log2 scale). Grey represent not expressed (RPKM = 0). **c**. Network analysis performed on differentially expressed genes between BC and CP revealed GLI2 as a key hub in the SHH pathway. **d**. Differentially expressed (DE) SHH genes at FDR 5% when comparing Blast crisis (BC) versus chronic phase (CP) or normal (CB and NPB). **e**. Box plots for GLI2 expression of 7 chronic phase (CP) and 6 blast crisis (BC) non-treated subjects, as well as 3 cord blood normal samples (CB) and 3 normal peripheral blood (NPB). Two-sided Jonckheere-Terpstra trend test: p = 0.014. **f**. G*LI1 and GLI2* transcripts were compared using quantitative RT-PCR in FACS-purified human cord blood and normal peripheral blood CD34^+^CD38^+^Lin^−^PI^−^ progenitor cells (n = 9, black), chronic phase CML (n = 7, blue) and in blast crisis CML (n = 10, red) patient derived samples. Values were normalized to RPL27 or HPRT housekeeping genes, and set to 1 for the normal progenitors. (Student’s *t*-test *p < 0.05).

To determine whether distinctive Hh pathway gene expression patterns predict CML progression, we utilized RNA-seq to compare expression levels of 41 a priori selected Hh pathway genes [19-21,45,46] in chronic phase (CP, n = 8), blast crisis (BC, n = 9), normal cord blood (CB, n = 3) and normal peripheral blood (NPB, n = 3) FACS-purified CD34^+^CD38^+^Lin^−^ progenitors (Additional file 1: Table S1 and S2). Principal component analysis (PCA) and unsupervised hierarchical clustering revealed distinctive expression level differences among 41 Hh pathway genes that significantly (p = 0.047, permutation test) separated normal from untreated CP and BC progenitors and BCR-ABL inhibitor treated samples (Figure 1a, b). The expression levels of 20,613 genes above detectable expression levels (RPKM > 0.2 in over 75% of samples) were compared between untreated CP (n = 7) and BC (n = 6) progenitors. Among 1,495 differentially expressed (DE) genes (FDR < 0.05), key SHH genes were included (Figure 1d). Notably, a GLI2 network was enriched in BC LSC for upregulated DE genes using Ingenuity software (Figure 1c). Both RNA Seq (Figure 1e) and qRT PCR (Figure 1f) analyses confirmed significantly increased expression of the SHH pathway transcriptional activator, GLI2, during BC transformation. Together these data suggest that critical regulatory components of the Hh signaling pathway, such as GLI2, represent part of a core transcriptional program driving human myeloid LSC maintenance for BC CML, thereby providing the impetus for testing the capacity of a selective SMO antagonist to eradicate LSC.

### Selective SMO inhibition reduces GLI2-expressing LSC burden in supportive niches

**Figure 2:**
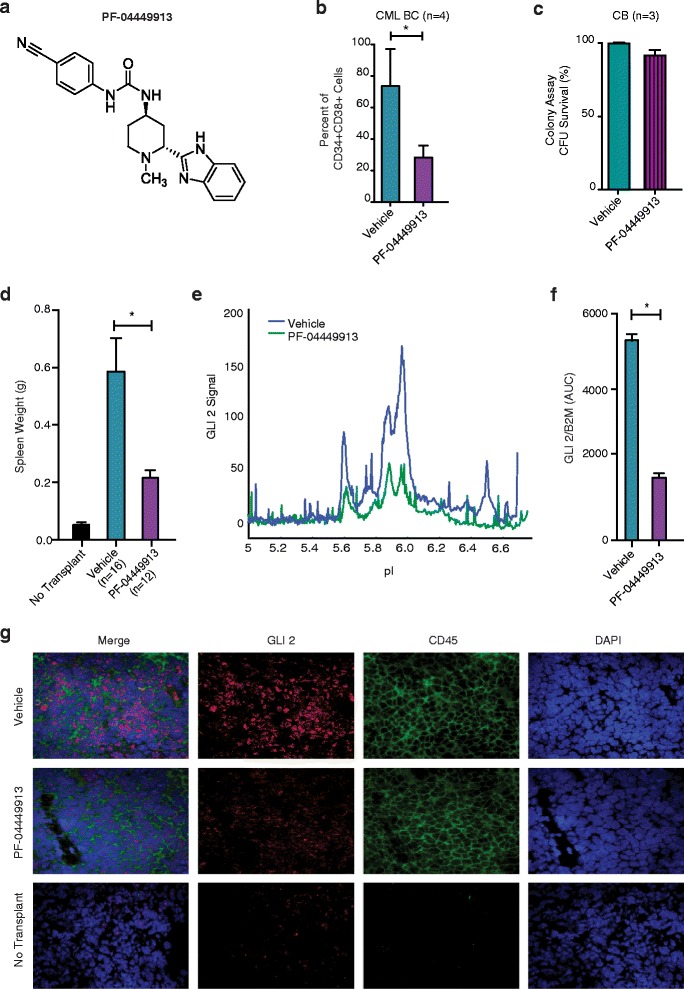
**Selective shh inhibition reduces lsc burden in stromal co-cultures. a**. Chemical structure of PF-04449913, a selective smoothened (SMO) antagonist. **b**. FACS analysis revealed a significant (Student’s *t*-test, *p = 0.047) reduction in blast crisis leukemic progenitor survival (n = 4 patient derived samples) following 7 days of PF-04449913 (1 μM, purple) compared with vehicle (DMSO, blue) treatment in SL/M2 co-cultures. **c**. Cord blood (n = 3) CD34^+^ cells were plated on SL/M2 co-cultures and treated with vehicle (DMSO) or PF-04449913 (1uM) for 7 days. Colony forming unit (CFU) survival was determined and compared to vehicle treatment. **d**. Spleen weight in blast crisis CML LSC engrafted mice after 14 days of treatment with vehicle (n = 16, blue) or PF-04449913 (n = 12; 100 mg/kg daily, purple). A significant (Student *t*-test, *p = 0.006) reduction is observed after PF-044449913 treatment. **e**. Nanoproteomic (CB1000) traces of total GLI2 protein of CD34 + CD38+ FACS sorted derived from the spleen of mice (n = 5) after vehicle (blue) or PF-04449913 (green) treatment for 14 days with 100 mg/kg daily. **f**. GLI2 expression was determined after normalizing the area under the curve (AUC) to a β2-microglobulin (β_2_M) loading control (Student’s *t*-test *p = 0.001) **g**. Confocal fluorescence microscopic analysis of spleen sections from no transplant or LSC engrafted mice treated with vehicle or PF-04449913. Photomicrographs of sections stained with DAPI and antibodies specific for human CD45, human GLI2 and the merged image.

A clinical small molecule SMO antagonist, PF-04449913 (Figure 2a), which was shown to compete for binding to human SMO (amino acids 181–787) with an IC50 of 4 nM (Additional file 1: Figure S1a), was utilized in this study, which is know to reduce GLI expression. PF-04449913 inhibited sonic hedgehog (Shh) stimulated luciferase expression in mouse embryonic fibroblasts with an IC50 of 6.8 nM (Additional file 1: Figure S1b, c); and significantly reduced medulloblastoma growth in a Ptch1^+/−^p53^+/−^ allograft model (Additional file 1: Figure S1d, e) at doses that decreased murine *Shh* target gene expression (Additional file 1: Figure S1f-h). To recapitulate extrinsic growth regulatory cues provided by the LSC niche, human BC CML progenitors were co-cultured on human SCF, IL-3 and G-CSF secreting (SL/M2) stromal layers. In stromal co-culture experiments, FACS analysis demonstrated a significant reduction in BC LSC (Figure 2b) by PF-04449913 when compared with normal progenitors and (Figure 2c). Importantly, human BC LSC engrafted RAG2^−/−^γ_c_^−/−^ mice treated daily with PF-04449913 compared with vehicle treated controls had a significant spleen weight reduction (p = 0.006) (Figure 2d). This reduction in leukemic burden corresponded with decreased GLI2 protein expression, as determined by both nanoproteomic analysis of FACS purified human progenitors (Figure 2e, f) and GLI2 confocal fluorescence microscopic analysis of splenic sections (Figure 2 g).

While some studies suggest that Hh signaling is dispensable for normal mouse hematopoiesis [40], others demonstrate that GLI regulates hematopoietic stem cell (HSC) fate decisions [47]. However, the role of Hh signaling in normal human HSC and progenitor cell maintenance had not been examined extensively in vitro or in primary sample xenograft models that permit robust engraftment. Thus, we compared the effects of PF-04449913 and vehicle treatment on normal hematopoietic stem and progenitor cells and demonstrated no change in differentiation capacity (Additional file 1: Figure S2a). When CD34^+^ cord blood engrafted NSG mice were treated with PF-04449913, the frequency of human CD45^+^ cells, progenitors and both myeloid and lymphoid cell fate commitment remained comparable to vehicle treated controls (Additional file 1: Figure S2b-d) indicating that unlike LSC, normal human HSC cell fate decisions are Hh pathway independent [41]. These results highlight the important niche dependent effects of selective SMO inhibition that induce GLI2 downregulation in a cell type and context specific manner.

### GLI2 induces cell cycle arrest in leukemic progenitors

**Figure 3:**
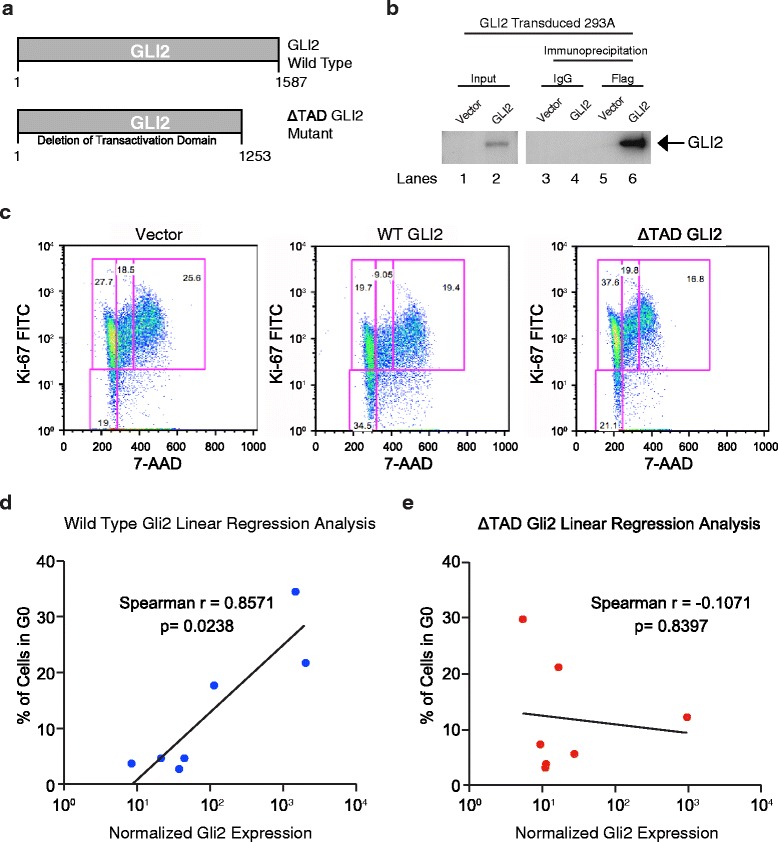
**GLI2 induces cell cycle arrest in leukemic progenitors. a**. Both the lentiviral GLI2 wild-type (GLI2) and transactivation domain deleted (GLI2 ΔTAD) expression constructs contain an N-terminus Flag epitope tag. The scheme depicts both wild type and mutant GLI2. GLI2 ΔTAD mutant harbors a deletion of the transactivation domain reported in [48]. **b**. Western blot of 293A cells transduced with GLI2 and immunoprecipitated using flag epitope. **c**. FACS based cell cycle analysis of patient samples (CD45+) that had either been transduced with vector, wild type (GLI2), or mutant (GLI2). **d-e**. Spearman correlation analysis comparing GLI2 expression levels, determined by quantitative PCR, and percent of cells in G0 after lentivital GLI2 or GLI2 ΔTAD compared with backbone vector controls.

Because LSC in the G0 phase of the cell cycle are considered dormant and typically resistant to therapies that target dividing cells and GLI transcriptional regulators have been implicated in cell cycle regulation, the role of GLI2 in LSC dormancy was examined. To this end, a lentiviral vector expressing full length human GLI2 was generated. As the key Hh pathway transcriptional activator, GLI2 is a 167 kDa protein that binds DNA through its zinc finger domain. To determine if GLI2 transcriptional activity is required to induce LSC dormancy, a GLI2 deletion mutant lacking the transactivation domain (GLI2ΔTAD) was generated (Figure 3a). Deletion of the transactivation domain of GLI2 inhibits GLI2 specific transcriptional activity [48]. In addition, both Western blot and immunoprecipitation analyses were used to validate GLI2 lentiviral vectors in 293A cells (Figure 3b). To determine if GLI2 induces LSC dormancy, chronic phase (CP) CML primary patient sample (n = 5) derived progenitors were transduced with backbone vector, GLI2, or GLI2ΔTAD lentiviral vectors and co-cultured on SL/M2 LSC supportive stroma [13]. To determine lentiviral GLI2 transduction efficiency in CML progenitors, Q-RT-PCR using GLI2 specific primers was employed. Multicolor cell cycle FACS analysis performed on these CML progenitors (Figure 3c) demonstrated an increase in the G0 population in samples transduced at high transduction efficiency with GLI2 compared to vector and GLI2ΔTAD. Notably, Spearman correlation analysis revealed a direct correlation between GLI2 expression levels and the percentage of cells in G0 (Figure 3d) in GLI2 transduced CP primary samples (n = 7, p = 0.024) in contrast to GLI2ΔTAD transduced samples, which did not expand the G0 fraction (n = 7; p = 0.84) (Figure 3e).

### SMO inhibition abrogates LSC dormancy in vivo

**Figure 4:**
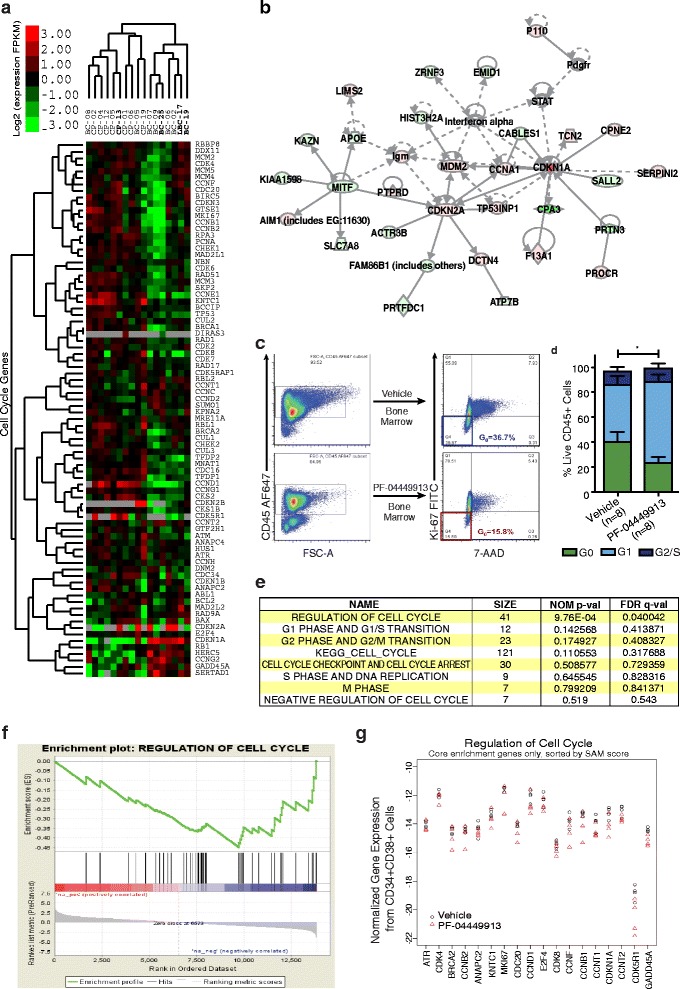
**Shh inhibition induces cycling of dormant leukemic progenitors. a**. Heatmap from patient samples, unsupervised agglomerative hierarchical clustering of cell cycle pathway genes using RNA-Seq data from FACS-purified progenitors (CD34^+^CD38^+^lin^−^PI^−^) from 8 chronic phase (CP) and 9 blast crisis (BC) patient samples. Bold indicates prior treatment before sample collection (Additional file 1: Table S1). Red indicates over- and green, under-expression relative to the median RPKM (Log2 scale). Grey represents no expression. **b**. Network analysis of BC and CP progenitors revealed CDKN1A and CDKN2A as cell cycle hub. **c**. Representative FACS plots in bone marrow CD45^+^ cells after 14 days of vehicle or PF-04449913 treatment. **d**. Cell cycle analysis of bone marrow from BC CML engrafted mice after 14 days of vehicle (n = 4) or PF-04449913 (n = 4). Student’s *t*-test *p < 0.05 for both G_0`_ and G_1_ population compared with vehicle treatment. **e**. GSEA analysis summary table obtained from RNA sequencing data comparing PF-04449913 treated engrafted mice (n = 4) to control (n = 4) (average 24.7-58.0 million mapped reads/sample). “Regulation of Cell Cycle” pathway was significantly decreased in PF-04449913 purified human progenitors derived from treated mice (family-wise p value =0.02). **f**. GSEA enrichment plot of “Regulation of Cell Cycle” pathway gene expression following in vivo LSC treatment with PF-04449913. The horizontal heatmap shows SAM score in descending order for all 13,850 genes (SHH pathway genes indicated as vertical black bars). **g**. Normalized gene expression values for the 18 genes in the core enrichment subset from the “Regulation of Cell Cycle” pathway. All the genes had a negative SAM score and are sorted in order of descending SAM score along the x-axis. This order agrees with the order in the GSEA enrichment plot, where expression levels for these genes are significantly reduced in the PF-04449913 treated mice.

To determine whether myeloid leukemic progression was associated with cell cycle pathway deregulation, we compared the expression patterns of cell cycle-related genes in FACS purified CP (n = 8) and BC (n = 9) progenitors. Unsupervised agglomerative hierarchical clustering analysis demonstrated that distinctive cell cycle gene expression patterns distinguished BC from CP samples (Figure 4a). Ingenuity pathway analysis revealed increased expression of cell cycle regulatory genes, such as CDKN1A and 2A, indicative of cell cycle arrest in BC progenitor LSCs (Figure 4b). Because dormancy gene induction could promote therapeutic resistance and relapse following TKI or chemotherapy discontinuation [49], we assessed the capacity of the SMO inhibitor, PF-04449913, to eradicate human BC LSC in xenograft studies. While PF-04449913 treatment increased the G1 fraction, it reduced the therapy resistant G0 fraction (Figure 4c, d). To elucidate a potential mechanism of cell cycle activation following selective SMO inhibition in vivo, RNA-seq was performed on surviving progenitors hematopoietic tissues that were FACS purified from hematopoietic tissues following treatment of human LSC engrafted mice (Additioanal file 1: Table S3). Significant repression of cell cycle regulators (family wise p-value 0.02) was observed in response to PF-04449913 treatment (Figure 4e-g). These experiments revealed that cell cycle induction in LSC represents a novel mechanism for the loss of LSC maintenance following SMO inhibition.

### GLI2 inhibition enhances LSC cycling and TKI sensitivity

**Figure 5:**
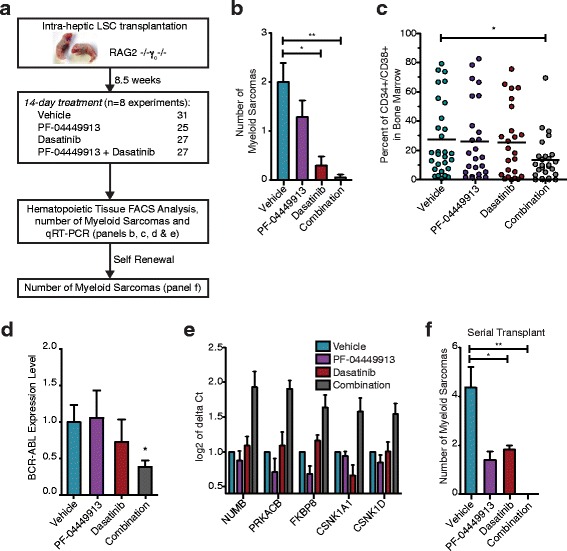
**PF-04449913 induced cell cycle activation enhances TKI sensitivity. a**. Schematic of in vivo experiments. RAG2^−/−^γ_c_^−/−^ pups were transplanted intrahepatically with 50,000 CD34^+^ BC CML cells within 48 hours of birth. Engrafted mice were treated daily for 14 days by oral gavage with vehicle, PF-04449913 (100 mg/kg), Dasatinib (50 mg/kg) or the combination. **b**. Graph of myeloid sarcoma count in blast crisis CML engrafted mice in vehicle (n =13, blue), PF-04449913 (n=7, purple), dasatinib (n =6, red) and combination (n =3, black) treated mice +/− SEM; *p < 0.05 and *p < 0.01 by ANOVA and Tukey post-hoc analysis **c**. FACS analysis showing percentage of marrow engrafted blast crisis progenitor LSC (n = 3 patients) after 14-day treatment with vehicle (n =31, blue), PF-04449913 (n =25, purple), dasatinib (n =27, maroon) and combination (n=27, grey). *p < 0.05 by ANOVA and Tukey post-hoc analysis **d**. BCR-ABL transcripts in the blast crisis CML engrafted marrow mice after 14 days of treatment. Graph shows normalized BCR-ABL expression (HPRT) +/− SEM; *p < 0.05 by ANOVA and Tukey post-hoc analysis **e**. Hedgehog pathway gene expression in FACS purified human progenitor cells from blast crisis LSC engrafted mouse marrow treated with vehicle (n = 3, blue), PF-04449913 (n = 4, purple) dasatinib (n = 4, maroon), combination (n=3, dark grey). Expression levels of seven genes were significantly altered by synergistic effect of PF-04449913 and Dasatinib (NUMB, PRKACB, CTNNB1, FKBP8, CSNK1A1, CSNK1D and STK36), where five represent SHH regulatory genes (graphed). **f**. Mice serially transplanted with FACS purified human progenitors from LSC engrafted mice treated with vehicle (n=12, green), PF-04449913 (n=12, purple), dasatinib (n = 8, maroon) or combination (n=7, grey) were examined for myeloid sarcomas; *p < 0.05 and *p < 0.01 by ANOVA and Tukey post-hoc analysis.

Having observed the reduction in dormancy following SMO inhibition, we investigated whether PF-04449913 sensitized BC LSC to dasatinib (Figure 5a) - a potent BCR-ABL TKI that targets dividing cells. Engrafted mice treated with a combination of PF-04449913 and dasatinib showed a significant reduction (p < 0.01, ANOVA) in myeloid sarcoma formation (Figure 5b) as well as in both bone marrow LSC burden (p < 0.05, ANOVA) (Figure 5c) and BCR-ABL1 (p < 0.05, ANOVA) expression (Figure 5d). Furthermore, quantitative RT-PCR Hh array analysis of FACS-purified progenitors revealed significantly (p = 0.05, Limma method) increased expression of negative Hh pathway regulators, including NUMB, PRKACB, FKBP8, CSNK1A1 and CSNK1D (Figure 5e) following combination therapy. Serial transplantation experiments demonstrated a significantly reduced capacity of LSC to form myeloid sarcomas (p < 0.01, ANOVA) following PF-04449913 and dasatinib therapy (Figure 5f). This study has provided the impetus for the on-going Phase 1b/ll combination SHH inhibitor and TKI inhibitor clinical trials for patients with advanced myeloid malignancies to both halt progression and prevent relapse.

## Discussion

RNA-Seq gene expression analyses of functionally validated LSC in advanced human myeloid malignancies, such BC CML, demonstrate that activation of stem cell self-renewal and survival pathways signifies therapeutic resistance that could translate into poor clinical outcomes. These studies suggest that LSC profiling may represent an important patient prognostication strategy. Current evidence also suggests that deregulated stem cell dormancy, survival and self-renewal pathway activation leads to malignant reprogramming of progenitors into LSC that drive therapeutic resistance, relapse and myeloid leukemia progression [2,7,15,50-52]. However, selective and clinically effective dormant human LSC eradication strategies have remained elusive.

Because SMO signals primarily through GLI2, we hypothesized that selective SMO inhibition would reduce the dormant LSC burden particularly when combined with TKIs. Indeed, SMO treatment enhanced TKI sensitivity and inhibited LSC self-renewal in stromal co-culture and LSC xenograft models at doses that spared normal hematopoietic stem and progenitor cells. Moreover, SMO inhibition enhanced BC LSC sensitivity to a potent BCR-ABL1 inhibitor, dasatinib, leading to a reduction in self-renewal capacity. These studies suggest that cell cycle induction in dormant LSC in conjunction with targeted therapy may prevent leukemic persistence and progression secondary to therapeutic resistance.

Other key hedgehog related mechanisms of chemotherapeutic resistance have been identified in other myeloid malignancies, such as AML. A recent study demonstrated that GLI alone is sufficient to drive UGT1A-dependent glucuronidation of Ara-C in AML thereby leading to drug resistance. This resistance was overcome by genetic or pharmacologic inhibition of GLI using a SMO antagonist [35]. Based on recent advances in leukemia clinical trials showing promise of a variety of molecular therapies, the key to eradicating an evolving population of cells lies in combination therapies that selectively inhibit multiple targets. Ideally, these targets will be specifically activated in the LSC compartment, and pharmacological inhibition will spare normal adult stem cells during the course of treatment.

While selective reduction in LSC self-renewal capacity provides strong pre-clinical evidence for a direct LSC inhibitory effect, the potential capacity of PF-04449913 to modify the malignant niche may also contribute to its anti-leukemic efficacy and will need to be examined in clinical correlative studies in ongoing clinical trials. Moreover, the clinical importance of SMO inhibitor mediated sensitization of dormant LSC to therapeutic modalities that target dividing cells will need to be assessed in Phase 2 clinical trials. Overall, our results inform the development of a clinically tractable strategy for obviating therapeutic resistance by selective SMO inhibitor targeting of dormant leukemia stem cells alone or in combination with standard of care in advanced hematologic malignancies.

## Conclusions

Our work addresses the role of smoothened (SMO) activation of its downstream hedgehog pathway transcriptional activator, GLI2, in human myeloid leukemia progression, leukemia stem cell self-renewal and dormancy. RNA-Seq, nanoproteomic and qRT-PCR analyses of FACS purified progenitors revealed that distinctive expression patterns of both SHH and cell cycle regulatory genes, including *GLI2cpg CDKN1A* and *CDKN2A*, were harbingers of myeloid leukemic progression and LSC generation. Because of the niche dependency of Hh pathway signaling, the capacity of a selective SMO antagonist, PF-04449913, to inhibit human LSC was evaluated in stromal co-cultures and in various hematopoietic tissues in xenografts models. In these systems, LSC burden decreased commensurate with GLI2 and cell cycle regulatory gene repression and while GLI2 overexpression enhances dormant human leukemia stem cell generation, selective inhibition of SMO abrogates it and sensitizes them to tyrosine kinase inhibitors. While lentiviral overexpression of human GLI2 enhanced CML progenitor dormancy in stromal co-culture assays, the transcriptionally inactive form of GLI2 did not. In addition, cell cycle FACS analysis revealed a reduction in dormant LSC following PF-04449913 treatment in all hematopoietic niches. Our data suggest that *GLI2* represents part of a core transcriptional program driving human myeloid LSC maintenance thereby providing the rationale for clinically assessing the capacity of a selective SMO antagonist alone or in combination with anti-proliferative agents to eradicate dormant therapy resistant LSC in advanced myeloid malignancies.

## Additional file

Additional file 1: Figure S1. PF-04449913 chemical properties and inhibition of shh signaling in a PTCH+/-P53+/- tumor model. **Figure S2.** SHH inhibition spares normal human hematopoietic progenitors. **Table S1.** Characteristics of patient banked samples used for preclinical studies. **Table S2.** Cytogenetics of banked primary patient samples used for RNA-Seq. **Table S3.** Characteristics of patient-derived xenografted samples analyzed by RNA-Seq.
