# Supplementary material for: GLI2 inhibition abrogates human leukemia stem cell dormancy
Source: J Transl Med. 2015 Mar 21;13:98. doi: 10.1186/s12967-015-0453-9 (PMC4414375; doi:10.1186/s12967-015-0453-9)
Supplement: Additional file 1: Figure S1. — PF-04449913 chemical properties and inhibition of shh signaling in a PTCH+/-P53+/- tumor model. Figure S2. SHH inhibition spares normal human hematopoietic progenitors. Table S1. Characteristics of patient banked samples used for preclinical studies. Table S2. Cytogenetics of banked primary patient samples used for RNA-Seq. Table S3. Characteristics of patient-derived xenografted samples analyzed by RNA-Seq. [file 12967_2015_453_MOESM1_ESM.pdf]

Supplementary Figure Legends.

SUPPLEMENTARY FIGURE 1. PF-04449913 CHEMICAL PROPERTIES AND INHIBITION OF SHH SIGNALING IN A PTCH$^{+/-}$P53$^{+/-}$ TUMOR MODEL

a. Competition-binding assay using a characterized cyclopamine-competitive SMO antagonist. PF-04449913 competes with the radiolabeled SMO antagonist for binding to human SMO (amino acids 181-787) with an IC50 of 4 nM (4.3 nM +/- 5.2 nM, N= 5). **b**. Inhibition of Shh stimulated luciferase expression using mouse embryonic fibroblasts expressing luciferase under control of an 8X GLI-response element (GLI-LUC MEFs). **c**. Dose dependent inhibition by PF-04449913 in the GLI-Luc MEF reporter assay; PF-04449913 inhibits Shh stimulated reporter activity with an IC50 of 6.8 nM (n=5). **d**. Anti-tumor activity of PF-04449913 against *Ptch+/-p53+/-* medulloblastoma. Allograft (~700 mm^3) bearing SCID-bg female mice were dosed orally once a day for six days with 100 mg/kg of PF-04449913 or vehicle. Tumor size (length and width) was measured using a caliper at regular intervals and tumor volume was calculated by standard procedure. Results are mean +/- standard error of the mean (n = 3 animals per group). **e**. Dose dependent anti-tumor efficacy of PF-04449913 against *Ptch+/-p53+/-* medulloblastoma allografts. Cohorts of allograft (~200 mm^3 to 1000 mm^3) bearing SCID-bg female mice were dosed orally once a day for six days with different dose levels of PF-04449913. Tumor size (length and width) was measured using a caliper at regular intervals and tumor volume was calculated by standard procedure. The percent change of an individual tumor volume was calculated from the tumor volume on the first day of dosing to the sixth day. Results are expressed as mean +/- standard deviation (n = 3 to 12 animals per group; $p < 0.01$ for 1 mg/kg group and $p < 0.001$ for all other groups). **f**. Genes significantly down-regulated by PF-04449913 treatment in *Ptch$^{+/-}$p53$^{-/-}$* mice. Hierarchical clustering was performed on Z-score transformed data using correlation similarity metric and centroid linkage method. **g**. Hh pathway inhibition in PF-04449913 treated *Ptch+/-p53+/-* medulloblastoma allografts. *Ptch+/-p53+/-*

allograft bearing SCID-bg female mice were dosed orally once a day for six days with 100 mg/kg of PF-04449913 or vehicle. Expression levels of *FoxM1, Gli1, Gli2, Mycn, Ptch1, Ptch2, Sfrp1* and *Smo* were determined by qRT-PCR. The vehicle-treated levels for each gene were normalized to 100%. **h**. Gene signatures enriched for within the top 31 PF-04449913-downregulated genes in *Ptch+/-p53 -/-* mice. Statistical significance of over-representation and observed/expected ratio were calculated as described in supplementary methods.

SUPPLEMENTARY FIGURE 2. SHH INHIBITION SPARES NORMAL HUMAN HEMATOPOIETIC PROGENITORS

a. Differentiation into CFU-Mix (purple), BFU-E (red), CFU-G (orange), CFU-M (yellow), CFU-GM (blue) of normal cord blood progenitors was assessed in hematopoietic progenitor assays (n=3) after PF-04449913 (1μM) or vehicle treatment for 14 days. **b.** FACS analysis was used to determine the total human $CD45^+$, hematopoietic stem and progenitor cell (HSPC), myeloid and lymphoid cell count in bone marrow after 14 days of treatment with vehicle (n=3, green) or 100 mg/kg of PF-04449913 (n=4, purple). **c**. FACS quantification of G0 (green), G1 (light blue) and G2/S (navy) human $CD45^+$ cells in cord blood engrafted marrow after 14 days of treatment with vehicle (n=3) or PF-04449913 100mg/kg (n=4). **d.** Representative FACS plots depicting HSPC, myeloid and lymphoid differentiation (panel B) in human cord blood engrafted mice after 14 days of treatment with vehicle or 100 mg/kg of PF-04449913.

SUPPLEMENTARY TABLE 1. Characteristics of patient banked samples used for preclinical studies.

SUPPLEMENTARY TABLE 2. Cytogenetics of banked primary patient samples used for RNA-Seq.

SUPPLEMENTARY TABLE 3. Characteristics of patient-derived xenografted samples analyzed by RNA-Seq

SUPPLEMENTARY METHODS

SMO RADIOLIGAND COMPETITION BINDING ASSAY

Membranes were prepared from a stable cell line created in HEK293FlpIn-TetR cells (Invitrogen) using Flp recombinase-mediated insertion of the pSecTag-FRT/V5-His vector containing a cDNA encoding amino acids 181-787 of human SMO fused to the murine Igk leader sequence to produce a cell surface expressed Smo 181-781 protein. LacZ-negative cells were analyzed for binding a well characterized cyclopamine-competitive tritiated Smo antagonist[40]. The tritiated ligand was prepared using Crabtree's catalyst and tritium gas. The labeled material was purified by RP HPLC (53.1 Ci/mmol specific activity at 99% purity). For the binding competition assay, 100μl of assay buffer was added to all the wells of a 96 well GF/B filter plate (Millipore MultiScreen-HTS-FB cat# MSFBN6B50). The plates were counted in a TopCount scintillation counter (Perkin Elmer). Data analysis uses Excel for % Inhibition and Graphpad Prism for IC_{50} calculation.

MOUSE EMBRYONIC FIBROBLAST GLI-LUCIFERASE ASSAY

Mouse Embryonic Fibroblasts expressing luciferase under control of an 8X Gli-response element (Gli-Luc MEFs)[41] were obtained from the Pfizer transgenic core facility. Luciferase activity was quantified with an EnVision plate reader (Perkin Elmer). Graphpad Prism was used for data analysis and IC_{50} calculation.

SELECTIVE SHH INHIBITION IN A MOUSE MEDULLOBLASTOMA ALLOGRAFT MODEL

Primary medulloblastoma tumors were harvested from *Ptch*$^{+/-}$*p53*$^{+/-}$ or *Ptch*$^{+/-}$*p53*$^{-/-}$ mice and propagated as allografts in SCID-bg mice 6-8 weeks of age (20 grams). Freshly isolated tumor fragments of approximately 50 mm^3 were surgically implanted subcutaneously into the hind flank region. Body weights and tumor size (length and width) were measured at regular intervals using a caliper and tumor volume was calculated using the formula: length (mm) x width (mm) x width (mm) x 0.4. For the tumor growth inhibition studies, cohorts of *Ptch*$^{+/-}$*p53*$^{+/-}$

medulloblastoma allograft bearing mice with tumors ranging from 200 mm^3 to 1000 mm^3 were dosed daily by oral gavage with vehicle (30% PEG 400/70% PBS) or with PF-04449913 formulated in vehicle.

Assessment of Target Gene Expression in Mouse Model: Microarray Processing

Microarray data were RMA normalized using Bioconductor Affy package. Differentially expressed genes were identified based on joint thresholds of t-test pvalue <0.01 and fold change >2. Their human orthologs were mapped using HomoloGene build 62. They were compared with curated gene sets from a variety of pathway/signature databases and enrichment P-value was determined using hypergeometric statistics calculated with Matlab. More specifically, the probability of observing at least (k) genes from a gene set is given by

$$P = 1 - \sum_{i=0}^{k-1} \frac{\binom{f}{i}\binom{g-f}{n-i}}{\binom{g}{n}}$$

where f is size of the gene set, n is the # of differentially expressed genes, (g) is the total number of unique human ortholog genes of mouse probes on the microarray. The obs/exp ratio was calculated as k/(f*n/g).

Supplementary Figure 1

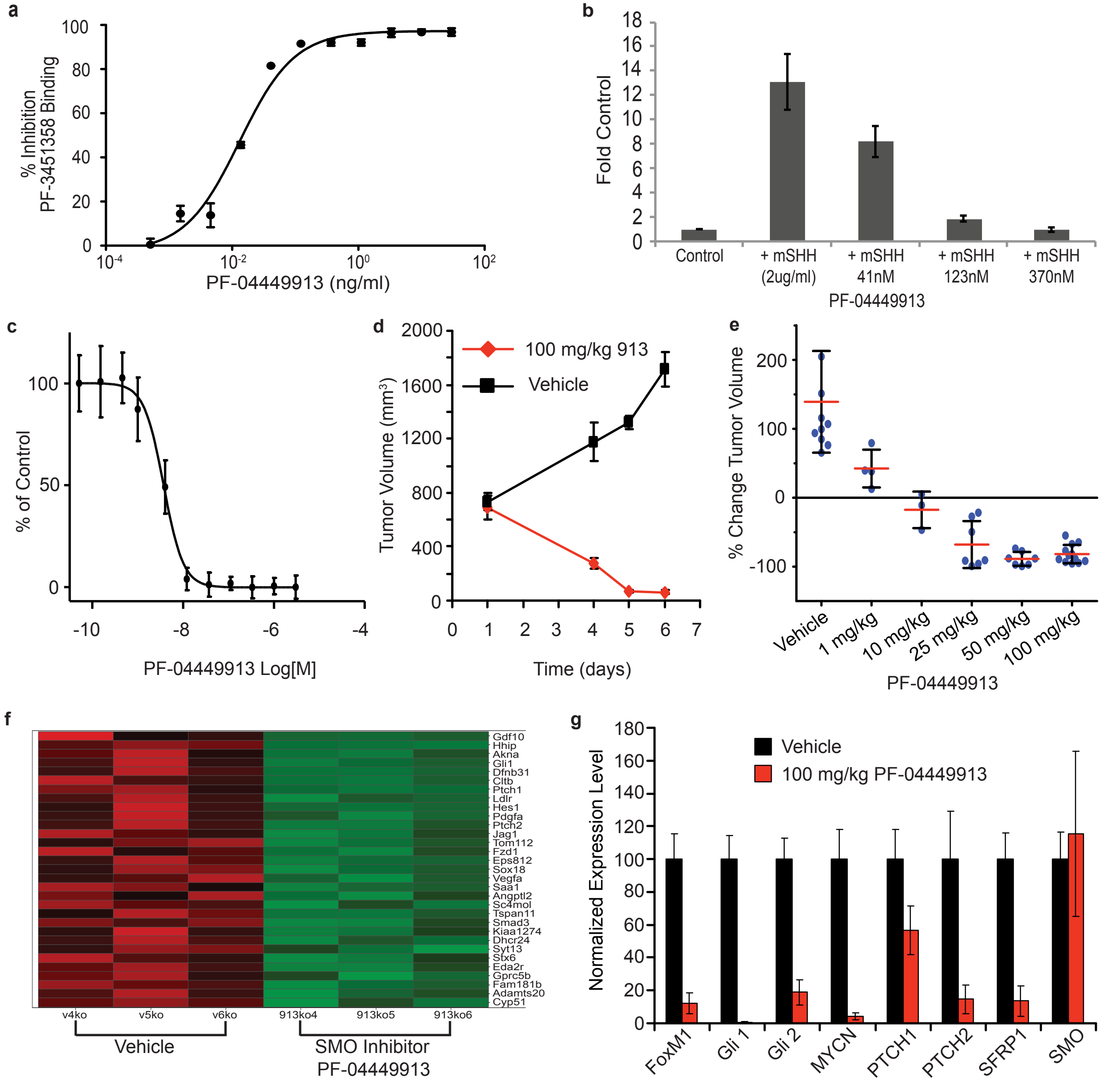

h

Signature	Source	P-Val	Obs/Exp
Hedgehog Up	NetPath	5.75E-10	114.42
Hedgehog Signaling Pathway	NetPath	7.26E-08	96.11
Sonic Hedgehog Signaling	Ingenuity	8.96E-08	91.53
Basal Cell Carcinoma	KEGG	9.00E-08	43.69
Basal Cell Carcinoma Signaling	Ingenuity	1.52E-07	39.39
SHH Gli Targets Neural Patterning Chip2 Vokes	msigDB	2.22E-07	73.93
Hedgehog Signaling Pathway	KEGG	4.18E-06	36.27
Axonal Guidance Signaling	Ingenuity	6.61E-06	9.48
Signaling Events Mediated by the Hedgehog Family	PID	1.22E-05	65.53
Epidermis Development	GO	1.51E-05	26.33
Multicellular Organismal Development	GO	1.71E-05	4.39
Biosynthesis of Steroids	KEGG	2.31E-05	53.29
Regulation of Smoothened Signaling Pathway	GO	4.17E-05	192.22
Platelet-derived Growth Factor Receptor Signaling Pathway	GO	6.25E-05	160.18
Smoothened Signaling Pathway	GO	6.25E-05	160.18

Supplementary Figure 2

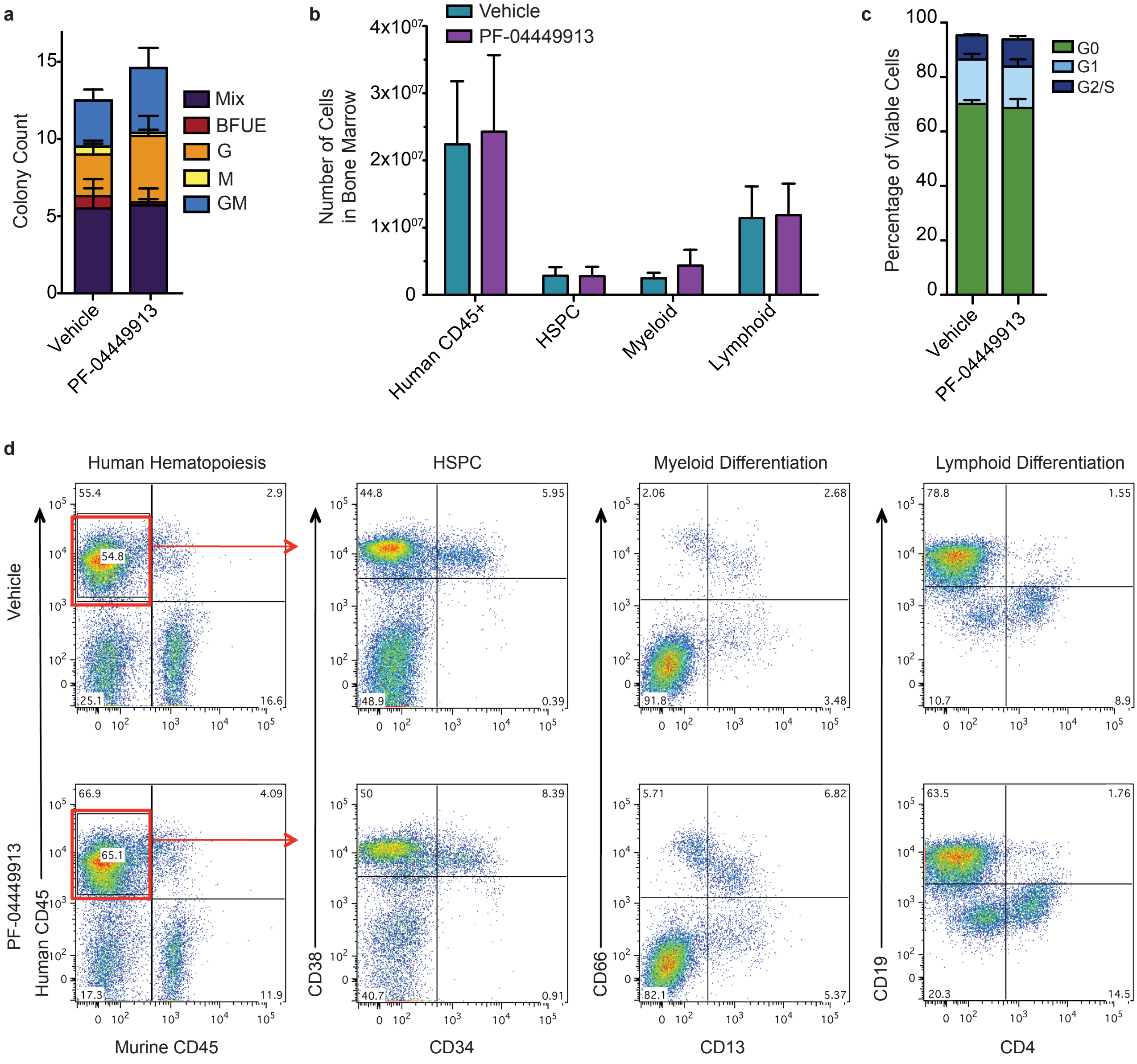

Supplementary Table 1

Characteristics of patient banked samples used for preclinical studies.

CML SAMPLES						
**Sample ID**	**Date**	**Sex/Age**	**Sample Type**	**WBC Count (K/mm^3)**	**% Blasts, PB**	**Treatment**
C001	26 Oct 07	N/A	Chronic Phase CML	N/A	N/A	N/A
C002	09 Jan 07	M	Chronic Phase CML	689	4%	Imatinib
C003	N/A	N/A	Chronic Phase CML	N/A	N/A	N/A
C004	05 Feb 07	F	Chronic Phase CML	N/A	N/A	N/A
C01	13 Nov 08	M/60	Chronic Phase CML	189	1.4%	None
C02	23 May 08	F/63	Chronic Phase CML	326	5%	None
C03	10 Dec 99	M/57	Chronic Phase CML	49	4.1%	None
C04	14 Oct 08	M/44	Chronic Phase CML	306	5.8%	None
C05	21 Sep 09	M/26	Chronic Phase CML	231	<1%	None
C06	25 Sep 09	F/68	Chronic Phase CML	88	<5%	Imatinib
C07	25 Mar 07	F/33	Chronic Phase CML	37.1	<1%	N/A
C08	29 Jan 99	M/56	Chronic Phase CML	381	<5%	Imatinib
C11	14 Jan 99	F/44	Chronic Phase CML	9.5	<1%	Hydroxyurea
C12	26 Aug 09	N/A	Chronic Phase CML	390	N/A	N/A
C13	21 Sep 07	F/50	Chronic Phase CML	N/A	N/A	Imatinib
B001	15 May 08	M/50	Blast Crisis CML	N/A	N/A	N/A
B002	N/A	N/A	Blast Crisis CML	N/A	N/A	N/A
B04	29 July 08	M/20	Blast Crisis CML	622	68%	Imatinib
B05	08 Dec 03	M/51	Blast Crisis CML	82.4	32%	Imatinib
B06	29 Oct 93	M/30	Blast Crisis CML	170	94.1%	Hydroxyurea
B07	29 Oct 93	M/48	Blast Crisis CML	209	86.1%	Hydroxyurea
B08	27 Jul 00	M/53	Blast Crisis CML	98	82.6%	Hydroxyurea
B09	17 Oct 91	M/65	Blast Crisis CML	72	41.7%	None
B10	21 Sep 93	M/40	Blast Crisis CML	133	82%	None
B11	16 Mar 06	M/31	Blast Crisis CML	40.1	79%	Hydroxyurea
B12	26 Jul 09	F/47	Blast Crisis CML	262	45%	Hydroxyurea
B13	16 May 08	M/49	Blast Crisis CML	8.4	15%	Imatinib
B14	16 Apr 04	M/40	Blast Crisis CML	47.7	47%	None
B15	8 Mar 05	F/31	Blast Crisis CML	11.4	55%	None
B16	22 Jul 02	F/52	Blast Crisis CML	60.3	14%	None
B20	N/A	?/37	Blast Crisis CML	N/A	N/A	N/A
B26	N/A	?/78	Blast Crisis CML	N/A	N/A	N/A

Supplementary Table 2

Cytogenetics of banked primary patient samples used for RNA seq.

Patient ID	Treatment	Cytogenetics	Immunophenotyping
CP-01	None	t(9;22)(q34;q11)	N/A
CP-02	None	46,XX,t(9;22)(q34;q11.2)[20], nuc ish(ABL1x3), (BCRx3), (ABL con BCRx2)[194/200]	N/A
CP-04	None	46,XY,t(9;22)(q34;q11.2)[20]	N/A
CP-05	None	46,XY,t(9;22)(q34;q11.2)[20]	N/A
CP-06	None	t(9;22)(q34;q11.2)	N/A
CP-12	None	N/A	N/A
CP-13	Imatinib	46,XX,t(9;22)(q34;q11.2), add(17)(p11.2~13) & T315I	CD34+ 6% of CD45+
CP-19	None	t(9;22)(q34;q11.2)	N/A
BC-02	None	t(9;22)(q34;q11.2)	CD11b 90%; CD13 99%; CD33 99%; CD34 88%; CD56 95%; HLA-DR 95%; CD10 21%; CD19 15%
BC-05	None	t(9;22)(q34;q11.2)	CD11b 95%; CD13 95%; CD33 95%; CD4 95%; CD117 48%; HLA-DR 90%
BC-06	Hydroxyurea	46,XY,t(9;22)(q34;q11.2)	CD13 70%; CD14 .28; CD33 74%; CD19 48%; HLA-DR 49%
BC-07	Hydroxyurea	46,XY,t(9;22)(q34;q11.2), add(18)(q?21).nuc ish 9q34(ABLx3),22q11(BCRx2)	CD13 76%; CD33 17%; CD19 90%; HLA-DR 88%; CD34 82%; CD10 84%
BC-08	Hydroxyurea	45,XY,-7,t(6;17;18)(p21.3;q23;p11.3), t(9;22)(q34;q11)	CD11 49%; CD13 100%; CD33 95%; CD56 91%; HLA-DR 92%; CD34 85%
BC-09	None	45,XY,-7, t(6;17;18)(p21.3;q23;p11.3), t(9;22)(q34;q11)	CD13 45%; CD14 68% CD33 67%; HLA-DR 58%
BC-17	Imatinib	t(9;22)(q34;q11.2)	CD34 46%; HLA-DR 83%; CD10 99%; CD19 99%; TdT 99%
BC-19	Imatinib followed by Dasatinib	t(9;22)-T315I	CD34+, CD13+, HLA-DR+, CD117+, MPO+, CD33+, weak CD79a, aberrant CD7+
BC-25	Imatinib & Hydroxyurea	T(9;22) and inv3(q21q26)	$CD117^{-}$, $HLA\text{-}DR^{-}$, CD33dim, CD38dim

Supplementary Table 3

Characteristics of patient-derived xenografted samples analyzed by RNA seq.

Sample #	Mouse #	# Cells	Treatment	RNA (ng/ul)	Amount (ng)	# Reads (Millions)
1	197	50k	Vehicle	15.06	225.9	96.9
2	197	23k	Vehicle	15.5	232.5	73
3	147	50k	Vehicle	16.91	253.65	21.4
4	147	50k	Vehicle	21.83	327.45	79.7
5	167	50k	Vehicle	27.12	406.8	109.8
6	167	50k	Vehicle	11.98	179.7	78.2
7	176	50k	SMO	16.46	246.9	88.3
8	176	30k	SMO	12.69	190.35	150.2
9	189	50k	SMO	13.54	203.1	157.9
10	191	50k	SMO	14.09	211.35	182.5
11	182	50k	Vehicle	14.05	210.75	187.1
12	187	50k	SMO	13.39	200.85	196.7
